# Supplementary material for: Evolution of fast-growing piscivorous herring in the young Baltic Sea
Source: Nat Commun. 2024 Dec 23;15:10707. doi: 10.1038/s41467-024-55216-8 (PMC11666761; doi:10.1038/s41467-024-55216-8)
Supplement: Supplementary file 1 — Supplementary Information [file 41467_2024_55216_MOESM1_ESM.pdf]

Evolution of fast-growing piscivorous herring in the young Baltic Sea

Jake Goodall[1], Mats E. Pettersson[1], Ulf Bergström[2], Arianna Cocco[1], Bo Delling[3], Yvette Heimbrand[2], Magnus Karlsson[4], Josefine Larsson[5], Hannes Waldetoft[4], Andreas Wallberg[1], Lovisa Wennerström[2], Leif Andersson[1,6*]

Supplementary Materials

Supplementary Figs 1-6

Supplementary Tables 1-11

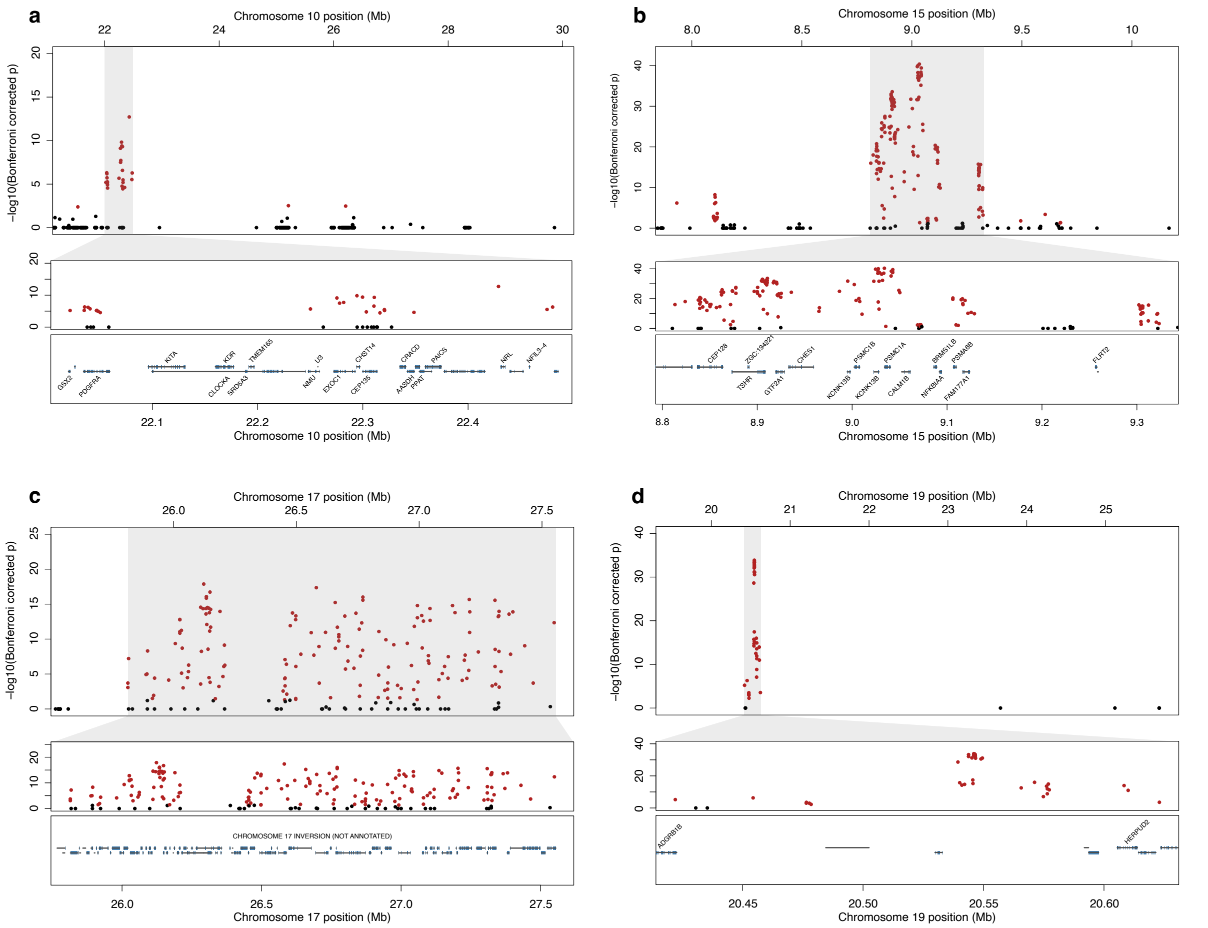

Supplementary Fig. 1. Summary of genome-wide, SNP-by-SNP, contrasts between spring-spawning Baltic herring and Slåttersill for the four main genomic regions on chromosomes 10 (**a**), 15 (**b**), 17 (**c**), and 19 (**d**). The topmost insets visualize chromosome-specific contrasts across the chromosome, while the centre and bottommost insets visualize a zoomed version of the peak region of interest (denoted by a grey box). Gene annotations intersecting with regions of interest are annotated in the bottommost inset, while points highlighted in red denote SNP with significant *P*-values (Bonferroni corrected $P<0.05$ from per SNP χ^2 test (d.f. = 1)). A list of all significant genes in the above plots is available from **Supplementary Data 1**. Chromosome 17 was not annotated as the region of interest spans the entire inversion region.

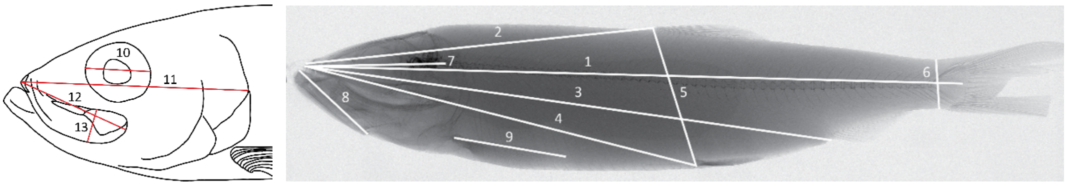

Supplementary Fig. 2. Measurements taken on *Clupea harengus* specimens; 1 standard length (SL), from upper jaw symphysis to middle base of caudal fin; 2, predorsal length from upper jaw symphysis to origin of dorsal fin; 3, preanal length, from upper jaw symphysis to origin of anal fin; 4, prepelvic length, from upper jaw symphysis to origin of pelvic fin; 5, body depth, origin of dorsal fin to origin of pelvic fins; 6, depth of caudal peduncle at origins of caudal fin procurrent fin rays; 7, cranium length, from upper jaw symphysis to articulation between the Atlas and next vertebra; 8, lower jaw length, from symphysis of dentary to retroarticular; 9, Pectoral fin length from base of first ray to tip of longest ray, digital calipers. 10, orbital horizontal diameter, x-ray; 11, head length, from upper jaw symphysis to posterior tip of operculum; 12, upper jaw length, from symphysis of premaxilla to posterior end of maxilla; 13, upper jaw depth, as greatest depth of maxilla and supramaxilla.

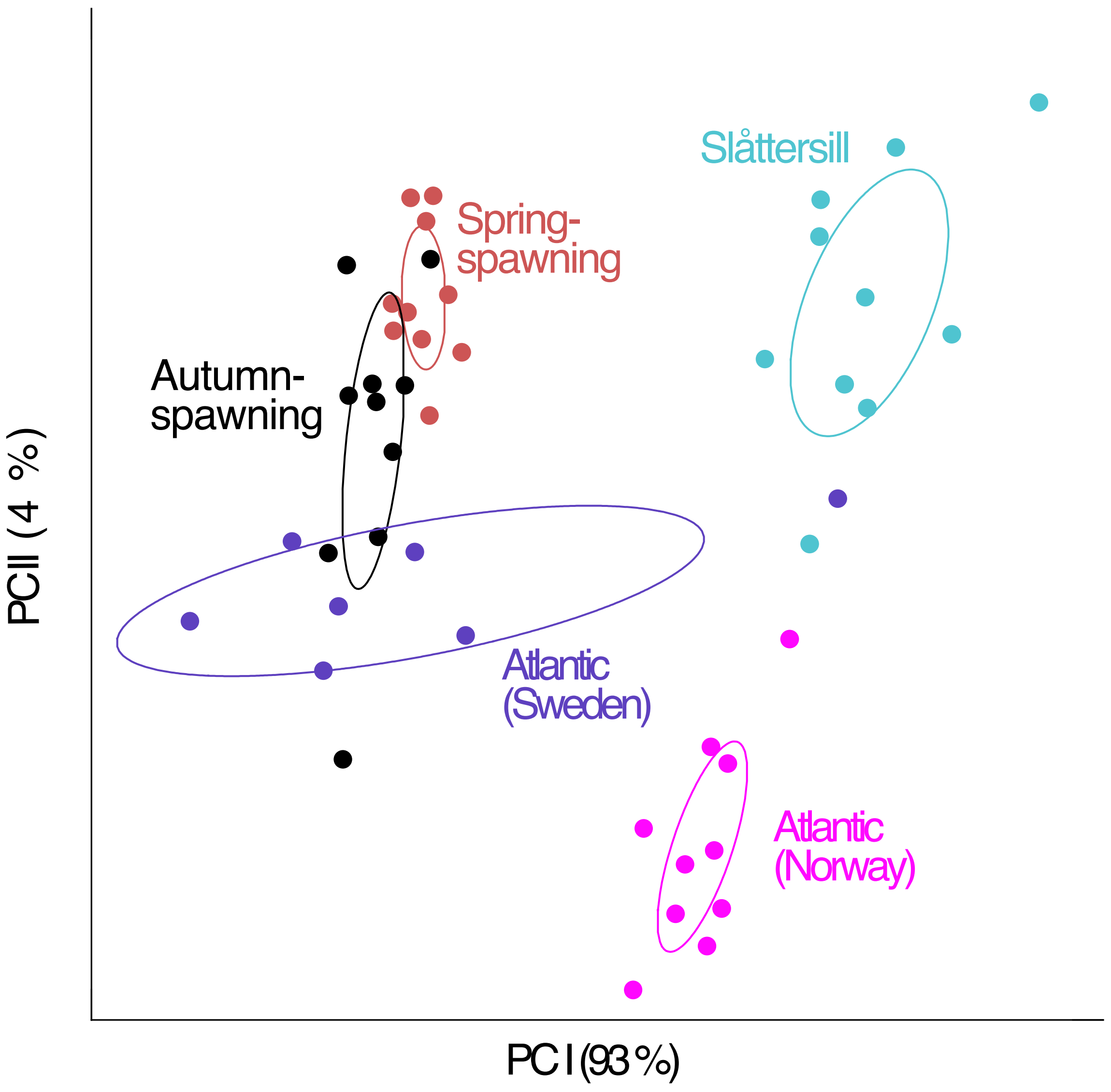

Supplementary Fig. 3. Principal component analysis of size and shape summarizing morphometric variation based on log-transformed measurement obtained from 47 specimens of *Clupea harengus* as shown in **Supplementary Data 2**. Corresponding character loadings are given in **Supplementary Table 3**. Ellipses indicate centroid positions with 95% confidence interval.

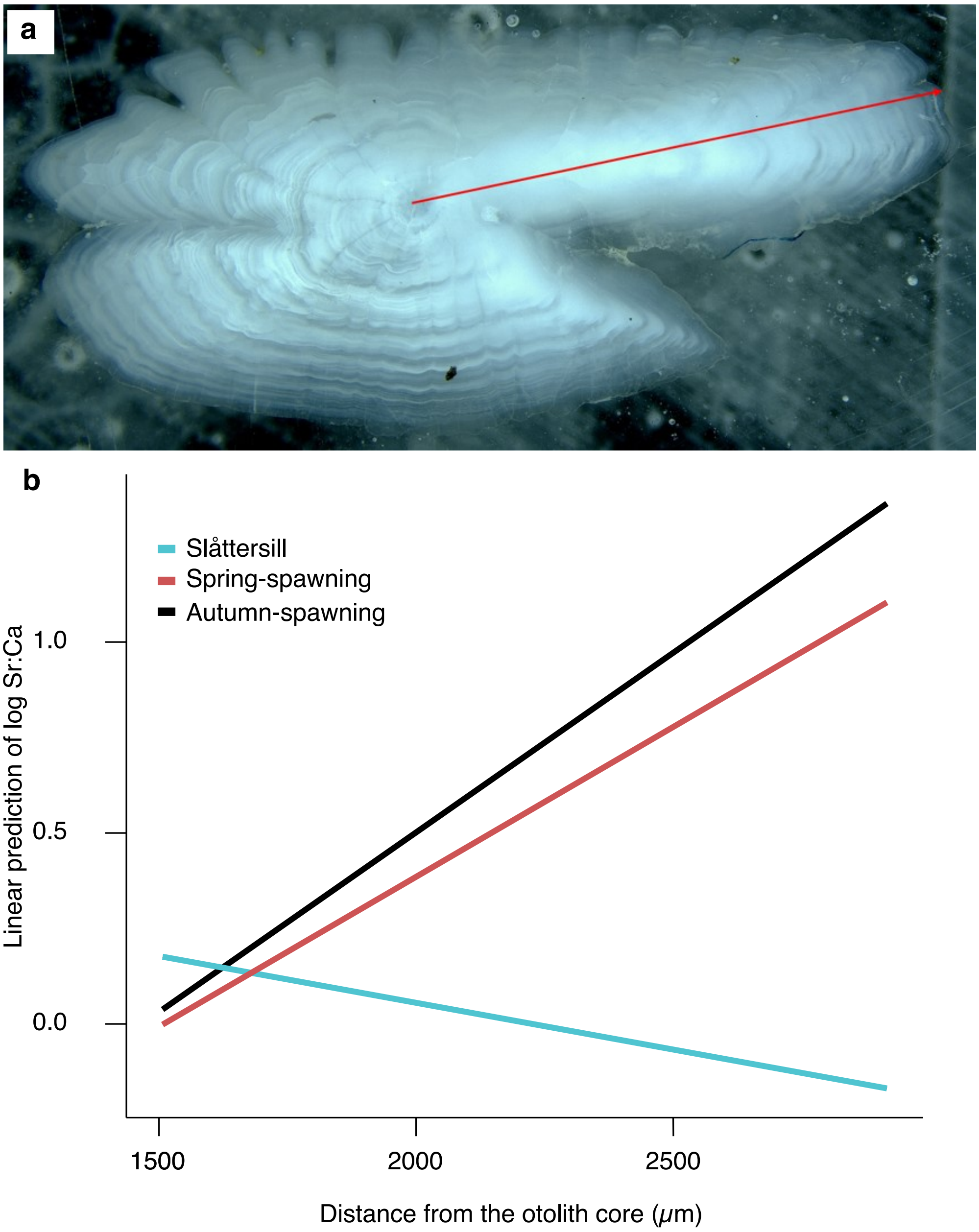

Supplementary Fig. 4. Results of otolith analysis. (**a**) Illustration of line transect (red line) from the otolith core to the anterior rostrum edge (top panel) used in lifelong otolith strontium:calcium profiling. (**b**) Pairwise comparison of otolith log transformed Sr:Ca slopes of Slåttersill (blue), autumn-spawning Baltic herring (black) and spring-spawning Baltic herring (red) from Gävlebukten. Only data from ≥ 1500 μm from the core were used in the analysis, corresponding to otolith material from adult herring only.

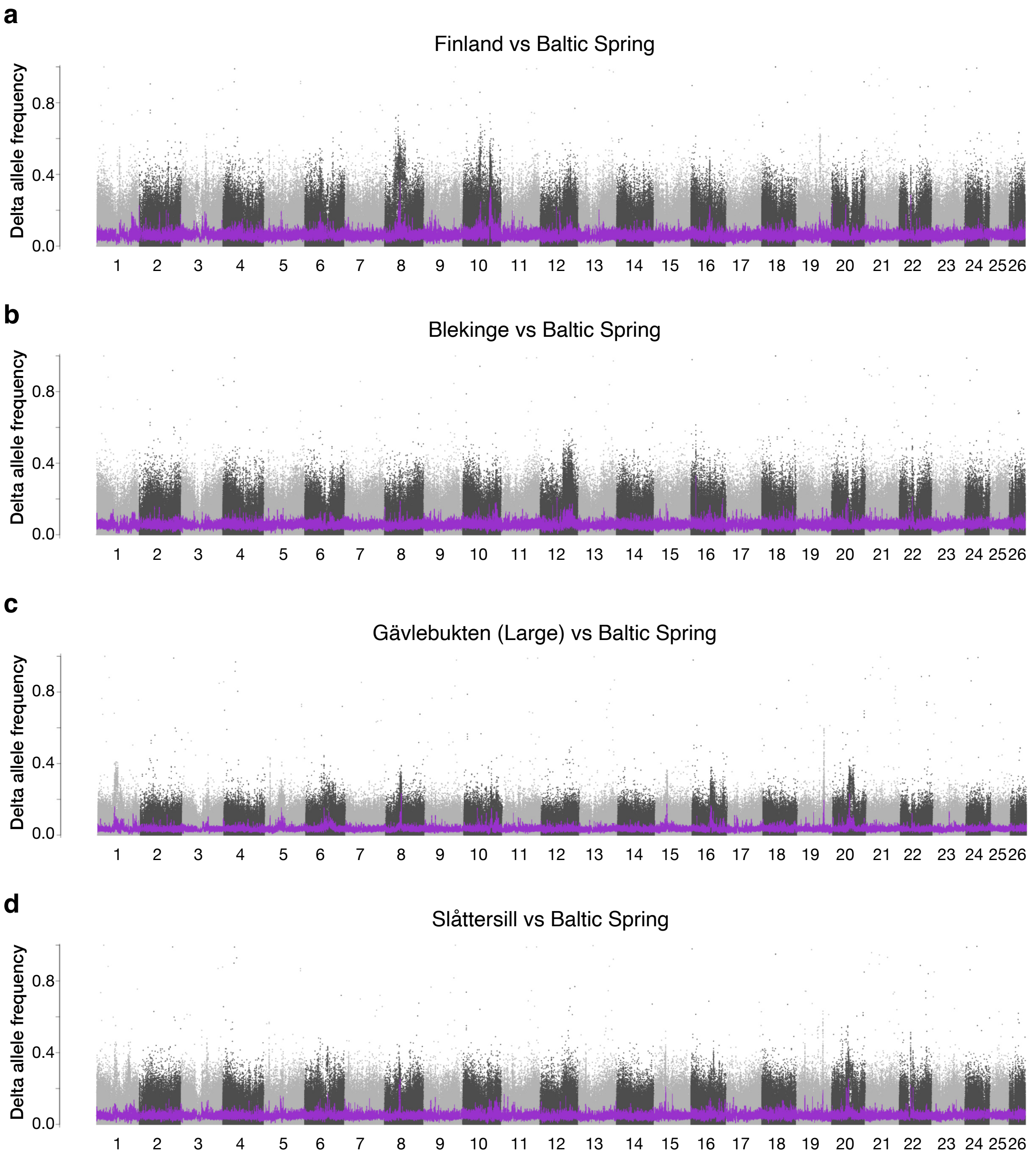

Supplementary Fig. 5 (continues on next page). Pairwise delta allele frequency contrasts between previously reported allele frequencies for small spring-spawning Baltic herring based on pooled whole genome sequencing[16] and various population samples of large herring based on individual whole genome sequencing in this study. (**a**) Finland (*n*=6), (**b**) Blekinge (*n*=7), (**c**) Gävlebukten (*n*=24), (**d**) Slåttersill (*n*=10), (**e**) Stockholm (*n*=16), (**f**) Östergötland (*n*=6), and (**g**) Kalmar (*n*=7). Notably, plots **a** – **d** represent large herring that exhibit a Northern haplotype at chr12, while plots **e** – **g** represent large herring with the Southern chr12 haplotype.

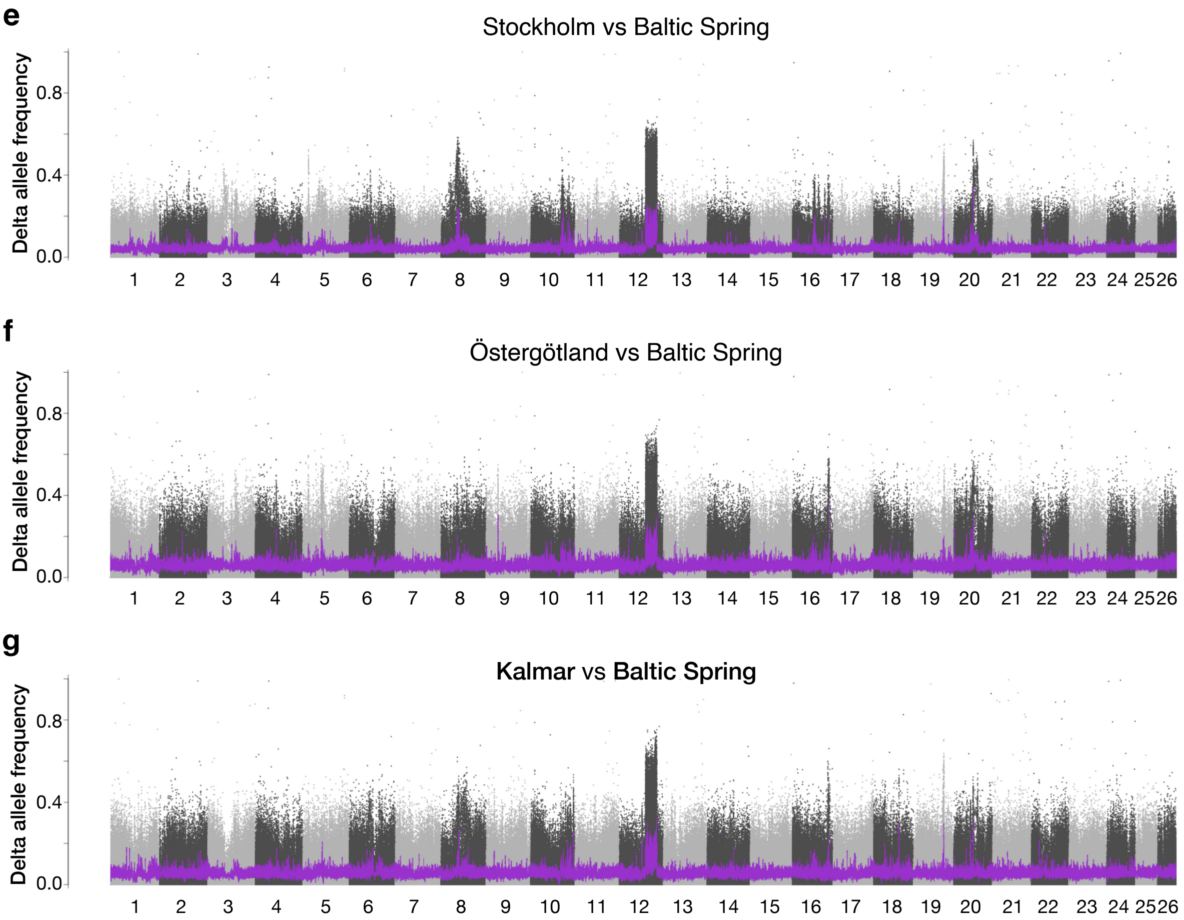

Supplementary Fig. 5. Pairwise delta allele frequency contrasts between previously reported allele frequencies for small spring-spawning Baltic herring based on pooled whole genome sequencing[16] and various population samples of large herring based on individual whole genome sequencing in this study. (**a**) Finland (*n*=6), (**b**) Blekinge (*n*=7), (**c**) Gävlebukten (*n*=24), (**d**) Slåttersill (*n*=10), (**e**) Stockholm (*n*=16), (**f**) Östergötland (*n*=6), and (**g**) Kalmar (*n*=7). Notably, plots **a – d** represent large herring that exhibit a Northern haplotype at chr12, while plots **e – g** represent large herring with the Southern chr12 haplotype.

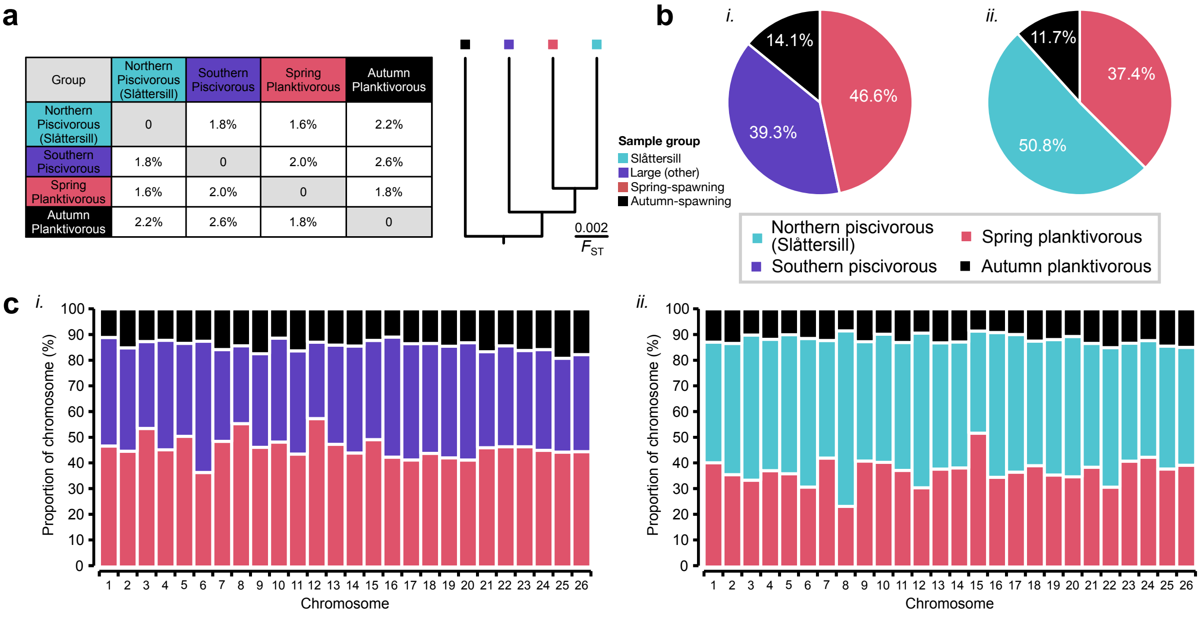

Supplementary Fig. 6. Pairwise genetic divergence among Baltic herring ecotypes. (**a**) Pairwise F_{ST}-matrix (left) and corresponding UPGMA clustering (right). Comparing northern piscivorous herring (*i*) and southern piscivorous herring (*ii*), respectively, the pie charts (**b**) show the overall proportion of 10-kbp windows across chromosomes 1–26 (*n*=79,406) with the lowest F_{ST} between each piscivorous population and each of the other ecotypes, and the stacked bar plots (**c**) show the corresponding signature on a chromosome-by-chromosome basis.

Supplementary Table 1. Summary of Baltic herring sampled from southern Bothnian Sea in 2022 and 2023 used for SNP genotyping on the MultiFishSNPChip_1.0 array (FSHSTK1D). Population number assignments are defined and displayed geographically in Fig. 1a.

Ecotype	Population	Location	Latitude	Longitude	Year sampled	Date sampled	Number of fish (n)	Average weight (g)	Average length (cm)
Slåttersill	1	Hästskär	60.532	17.730	2022	2022-06-16	30	192.0	28.5
Slåttersill	1	Hästskär	60.532	17.730	2023	2023-06-15	78	181.0	27.8
Spring-spawning	2	Stocka	61.540	17.280	2022	2022-04-13	30	38.3	18.7
Autumn-spawning	3	Eggegrund	60.737	17.561	2022	2022-10-19	30	51.0	19.9
Autumn-spawning	4	Fagerviken	60.542	17.741	2022	2022-09-05	30	49.3	19.5
Spring-spawning	5	Hästskär	60.532	17.730	2022	2022-06-01	30	55.7	21.0
Spring-spawning	5	Hästskär	60.532	17.730	2023	2023-06-18	60	55.8	20.0
Spring-spawning	6	Norrsundet	60.936	17.372	2022	2022-06-14	30	47.7	20.0
Spring-spawning	7	Lötbäcken	61.250	17.110	2022	2022-05-23	30	46.4	19.9
Autumn-spawning	8	Limön	60.410	17.350	2022	2022-09-05	36	41.3	18.2

Supplementary Table 2. Summary of age estimates based on the analysis of otoliths (Spring-spawning Baltic herring: $\bar{x}$-$\bar{y}$ = 3.42 years, *C.I.* = 2.06 – 4.78 years, two-sided t-test, $t(49) = 5.06$, $P = 6.4 \times ^{-8}$; Autumn-spawning Baltic herring: $\bar{x}$-$\bar{y}$ = 2.48 years, *C.I.* = 1.34 – 3.61 years, two-sided t-test, $t(49) = 4.39$, $P = 6.0 \times ^{-8}$).

		Age at capture	
	Number of fish (n)	Mean (± SD)	*P* *
Slåttersill	31	7.2 (± 3.4)	-
Spring-spawning Baltic herring	31	10.1 (± 3.2)	0.001
Autumn-spawning Baltic herring	26	9.2 (± 2.3)	0.006

Supplementary Table 3. Character loadings on principal component I-V for 13 morphological measurements taken on 47 specimens of *Clupea harengus*.

	I	II	III	IV	V
Standard length	0.183	-0.014	0.004	0.002	0.013
Orbital horizontal diameter	0.087	0.055	-0.017	0.000	0.009
Head length	0.150	0.020	0.005	-0.018	-0.008
Cranium length	0.136	0.053	-0.012	0.012	-0.001
Body depth	0.239	-0.047	-0.035	0.011	-0.024
Caudal peduncle depth	0.216	-0.060	0.013	-0.005	0.014
Predorsal length	0.183	-0.006	-0.001	-0.001	0.012
Preanal length	0.189	-0.009	0.001	0.009	0.008
Prepelvic length	0.180	0.005	0.001	0.012	0.003
Lower jaw length	0.147	0.045	0.003	0.007	0.008
Upper jaw length	0.148	0.034	-0.002	-0.014	-0.009
Upper jaw height	0.136	0.013	0.051	0.012	-0.019
Pectoral fin length	0.174	0.003	-0.001	-0.030	-0.004
Variance explained by components	0.380	0.016	0.004	0.002	0.002
Percent of total variance explained	93.3	3.9	1.1	0.5	0.4

Supplementary Table 4. Summary of biochemical comparison of Slåttersill (5 pools, *n*=98 fish total) and spring-spawning Baltic herring (9 pools, *n*=170) sampled from Gävlebukten across 2022 and 2023. All *P* values were derived from two-sided t-tests comparing Slåttersill and spring-spawning Baltic herring.

	Mean (± SD)		
	Slåttersill	Spring-spawning Baltic herring	*P*-value
Pollutant analysis			
Fat content (%)	10.0 (± 2.1)	3.9 (± 0.6)	0.002
∑PCDD/F (pg TEQ/g ww)	2.2 (± 0.5)	5.2 (± 0.8)	<0.001
∑PCDD/F + dl-PCB (pg TEQ/g ww)	5.2 (± 1.0)	8.6 (± 1.7)	0.001
∑PCB6 (ng/g ww)	33.2 (± 7.3)	40.4 (± 6.4)	0.104
Isotope analysis			
N (%)	9.9 (± 0.9)	11.8 (± 1.0)	0.005
δ15N (‰)	10.5 (± 0.3)	10.4 (± 0.4)	0.68
F_N (%)	0.4 (± 0.0)	0.4 (± 0.0)	0.524
C (%)	57.2 (± 1.7)	53.2 (± 2.7)	0.005
δ13C (‰)	-24.3 (± 0.6)	-22.8 (± 0.7)	0.002
F_C (%)	1.1 (± 0.0)	1.1 (± 0.0)	0.002
C/N ratio	5.8 (± 0.7)	4.5 (± 0.6)	0.008

Supplementary Table 5. Results of pairwise comparison of otolith log transformed Sr:Ca slopes (≥ 1500 µm from the core) of Slåttersill, spring- and autumn spawning herring from Gävlebukten based on estimated marginal means from a mixed effects model (**Supplementary Fig. 4b**). CL = confidence level.

Pairwise comparison	Estimate	SE	df	Lower CL	Upper CL	T ratio	*P* value
Slåttersill - Autumn-spawning	0.0012	4.53e-05	1731	0.001	0.001	26.147	<0.001
Slåttersill - Spring-spawning	-0.0010	2.38e-05	1718	-0.001	-0.001	-43.169	<0.001
Autumn-spawning - Spring-spawning	0.0001	4.89e-05	1730	0.000	0.000	3.185	0.004

Supplementary Table 6. Summary of large herring and control sample (Gävleborg, Norrsundet) sampled from the Baltic Sea for whole genome sequencing. Population number assignments are defined and displayed geographically in Fig. 1a.

Ecotype	Population	Locality	Location	Date sampled	Number of fish (n)	Average weight (g)	Average length (cm)
Slåttersill	1	Uppland	Hästskär	2022-06-16	10	254.0	30.7
Large	9	Blekinge	Karlskrona	2020-09-16, 2020-11-07	7	86.6	23.4
Large	10	Finland	Helsinki	2021-03-23	6	423.3	35.8
Large	11	Gävleborg	Storjungfrun	2020-10-27, 2020-10-28	14	159.9	27.4
Large	11	Gävleborg	Stations	2020-11-05	4	203.8	29.2
Large	11	Gävleborg	Iggön	2020-10-30, 2020-08-20	6	64.5	21.0
Large	12	Kalmar	Västervik	2020-10-30	7	72.9	22.4
Large	13	Östergötland	Birkö	2020-09-22	6	62.0	19.8
Large	14	Stockholm	Ornö	2020-11-30, 2020-09-30	16	165.8	27.0
Spring-spawning	15	Gävleborg	Norrsundet	2022-06-14	10	48.0	20.0

Supplementary Table 7. Levels of genetic variation across the genome (chromosomes 1 to 26) and estimated effective population. The analysis is based on the assumption that all sites along chromosomes were accessible (n=724,810,791 bp). Long-term inference of effective population size (N_e) estimated assuming mutation-drift equilibrium in an idealized population.

Group	Sample size (n)	SNPs (n)	θ_W/bp	π/bp	Tajima's D	N_e (n)
Spring planktivorous[1]	80	16,944,883	0.0047	0.21	-1.92	590,007
Autumn planktivorous[2]	60	15,266,334	0.0045	0.22	-1.84	564,593
Northern piscivorous[3]	68	17,268,431	0.0050	0.24	-1.86	621,815
Southern piscivorous[4]	58	15,098,960	0.0045	0.23	-1.78	562,528
