# Supplementary material for: Evolution of fast-growing piscivorous herring in the young Baltic Sea
Source: Nat Commun. 2024 Dec 23;15:10707. doi: 10.1038/s41467-024-55216-8 (PMC11666761; doi:10.1038/s41467-024-55216-8)
Supplement: Supplementary file 2 — Description of Additional Supplementary Files [file 41467_2024_55216_MOESM2_ESM.pdf]

Description of Additional Supplementary Files

File Name: Supplementary Data 1
Description: Catalogue of all significant loci (Bonferroni corrected $P < 0.05$; χ^2 test) in a SNP-by-SNP contrast between Slåttersill and spring-spawning Baltic herring.

File Name: Supplementary Data 2
Description: Samples of Atlantic and Baltic herring included in the morphological analyses. Summary of the analysis of meristic characters. =

File Name: Supplementary Data 3
Description: Full list of SNP designs for the MultiFishSNPChip_1.0 array (FSHSTK1D). The seventyonemer column illustrates the target SNP (enclosed within square bracket) for a given position, and its flanking regions which may contain additional known SNPs (enclosed in round brackets). The chromosomal positions refer to *Clupea harengus* reference assembly (Ch_v2.0.2.fasta).

File Name: Supplementary Data 4
Description: Summary of sample groups used for analyses of pairwise genetic divergence among Baltic ecotypes (Supplementary Fig. 6) and genome level estimations of genetic variation and effective population size (Supplementary Table 7).
