# Supplementary material for: Evolution of fast-growing piscivorous herring in the young Baltic Sea
Source: Nat Commun. 2024 Dec 23;15:10707. doi: 10.1038/s41467-024-55216-8 (PMC11666761; doi:10.1038/s41467-024-55216-8)
Supplement: Supplementary file 8 — Source data [file 41467_2024_55216_MOESM8_ESM.zip › Source_Data/Figure_2B.pdf.zip!/Figure_2B.pdf]

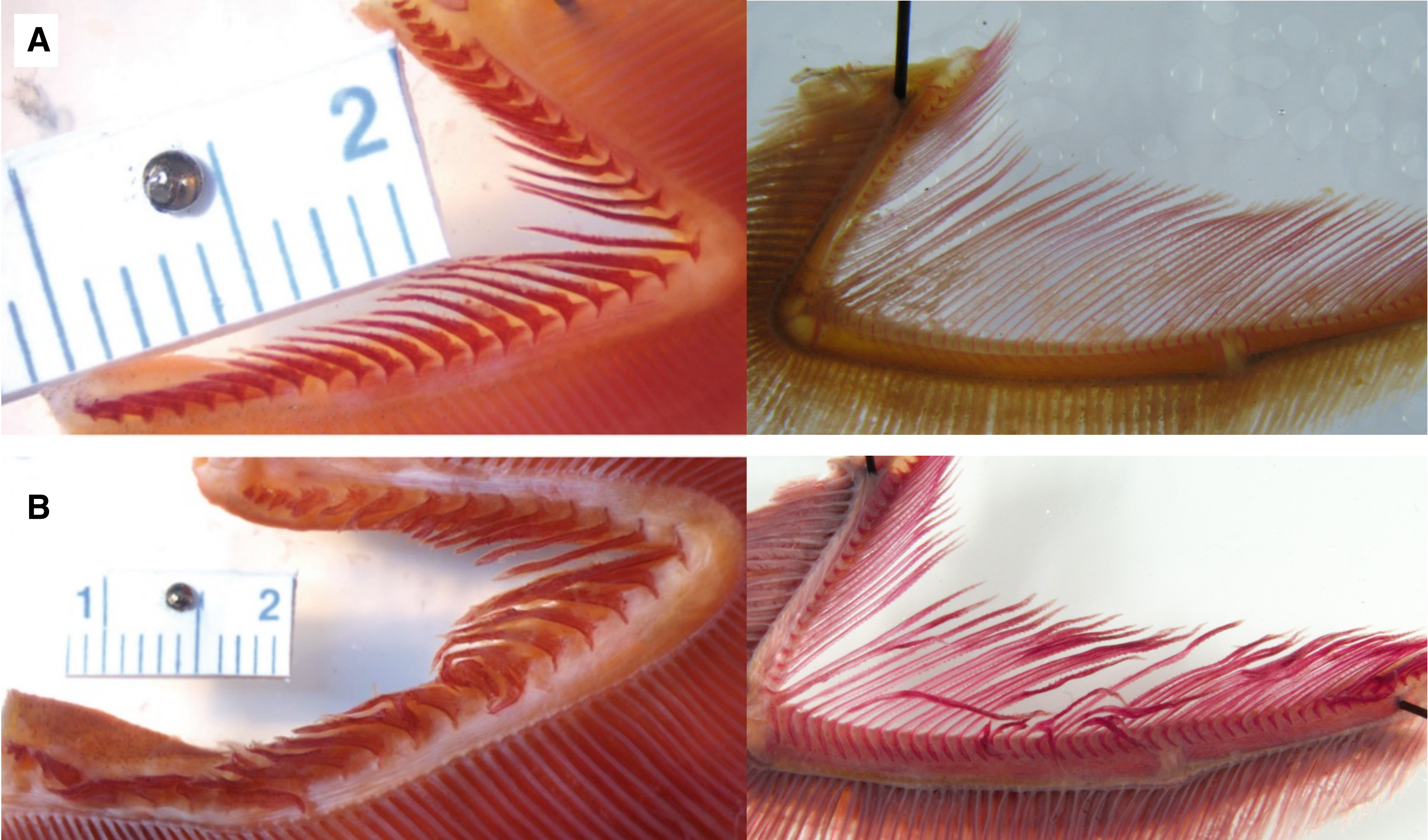

A
2
B
1
2
